# Information Geometry of the Exponential Family of Distributions with Progressive Type-II Censoring

**DOI:** 10.3390/e23060687

**Published:** 2021-05-28

**Authors:** Fode Zhang, Xiaolin Shi, Hon Keung Tony Ng

**Affiliations:** 1 Center of Statistical Research, School of Statistics, Southwestern University of Finance and Economics, Chengdu 611130, China; fredzh@swufe.edu.cn; 2 School of Electronics Engineering, Xi’an University of Posts and Telecommunications, Xi’an 710121, China; linda20016@163.com; 3 Department of Statistical Science, Southern Methodist University, Dallas, TX 75275-0332, USA

**Keywords:** information geometry, exponential family of distributions, progressively type-II censoring scheme, Bayesian prediction, asymptotic expansions, 62N05, 62C10, 62E20, 53B05

## Abstract

In geometry and topology, a family of probability distributions can be analyzed as the points on a manifold, known as statistical manifold, with intrinsic coordinates corresponding to the parameters of the distribution. Consider the exponential family of distributions with progressive Type-II censoring as the manifold of a statistical model, we use the information geometry methods to investigate the geometric quantities such as the tangent space, the Fisher metric tensors, the affine connection and the α-connection of the manifold. As an application of the geometric quantities, the asymptotic expansions of the posterior density function and the posterior Bayesian predictive density function of the manifold are discussed. The results show that the asymptotic expansions are related to the coefficients of the α-connections and metric tensors, and the predictive density function is the estimated density function in an asymptotic sense. The main results are illustrated by considering the Rayleigh distribution.

## 1. Introduction

From the geometrical viewpoint, a parametric statistical model can be considered a differentiable manifold, and the parameter space can be regarded as a coordinate system of the manifold [1,2]. Let F={f(x;θ),θ∈Θ} be a parametric statistical model with respect to some σ-finite reference measure μ, where θ is a real *k*-dimensional parameter vector belonging to some open subset Θ of the *k*-dimensional real space $R^{k}$. For simplicity, a random variable *X* and its observed value *x* are uniformly denoted by *x* in this paper.

When the density function f(x;θ) is sufficiently smooth in θ and it is differentiable as a function of θ, it is natural to introduce the structure of an *k*-dimensional manifold in the statistical model F, where θ plays the role of a coordinate system. The geometrical quantities, such as connection, divergence, flatness, curvature and tangent space, play a fundamental role in the statistical inference and asymptotic theory (see, for example, Komaki [3,4] and Harsha and Moosath [5]).

In reliability engineering, a life testing experiment is one of the effective ways to obtain reliability information of a product. To save time and reduce the cost of a life testing experiment, censoring methodologies are often applied so that the experiment is terminated before all the items on the life-testing experiment fail. Some commonly used censoring schemes include the Type-I and Type-II censoring schemes, where the life-testing experiment will be terminated at a prefixed time point and the life-testing experiment will be terminated as soon as the *m*-th (*m* is prefixed) failure is observed, respectively. In other words, the experimental time is prefixed for Type-I censoring scheme and the number of observed failures is prefixed for the Type-II censoring scheme (see, for example, Ng [6]). The Type-I and Type-II censoring schemes have been generalized to a more complicated and flexible censoring scheme such as progressive censoring schemes [7,8,9] and hybrid censoring schemes [10,11]. For progressive Type-II censoring schemes, the conventional Type-II censoring scheme is extended to situations wherein censoring occurs in multiple stages. A progressive Type-II censored life-testing experiment will be carried out in the following manner. Suppose *n* items are placed on a life-testing experiment and we assume that these *n* items have lifetimes following distribution with density function f(x;θ). It is planned that *m* failures will be observed and $R_{r}$ items are randomly removed (i.e., censored) from the experiment at the time of the *r*-th failure. More specifically, at the time of the first failure (denoted by $X_{1:m:n}$), $R_{1}$ randomly selected items from the n−1 surviving items are removed from the life testing experiment; then, the experiment continues and at the time of the second failure (denoted by $X_{2:m:n}$), $R_{2}$ randomly selected items from the $\left(n-R_{1}-2\right)$ surviving items are removed from the experiment, and so on; finally, at the time of the *m*-th item failure (denoted by $X_{m:m:n}$), the experiment terminates and all the remaining $R_{m}=n-m-\sum_{r=1}^{m-1}R_{r}$ surviving items are censored. Here, $R=(R_{1},R_{2},\ldots ,R_{m})$ is the progressive Type-II censoring scheme for the life testing experiment with $\sum_{r=1}^{m}R_{r}=n-m$. Note that, when $R_{1}=R_{2}=\ldots =0$, $R_{m}=n-m$, the progressive Type-II censoring scheme reduces to the conventional Type-II censoring scheme.

Since the comprehensive studies of information geometry by Amari [1], information geometry has been productively used in different research fields including statistical learning, machine learning, neural networks, signal processing, information theory and so on (see, for example, Amari et al. [2] and Amari [12].) The information geometry methods are also widely used in statistics and reliability engineering. For example, Zhang et al. [13] discussed the Amari-Chentsov structure on the accelerated life test model with applications to optimal designs with different optimal criteria. The methods of information geometry are also employed to investigate the Bayesian prediction by taking α-divergences as loss functions [14]. In degradation modeling, a robust parameter estimation method was proposed in [15] by minimizing the *f*-divergence between the true model and suggested models.

In this paper, we investigate the tangent space, affine connection, α-connection, torsion and Riemann-Christoffel curvature of the manifold of the exponential family of distributions with progressive Type-II censoring scheme. These geometric quantities can be applied to different areas in statistics such as Bayesian analysis. Note that one of the challenges of Bayesian analysis is to calculate the integrals for obtaining the posterior distribution, especially when the number of parameters is large. Instead of using numerical methods to approximate those integrals, the geometric quantities developed in this paper can provide an efficient theoretical method to approximate those integrals involved in Bayesian prediction. The main contributions and the organization of this paper are described as follows:

- Asymptotic theory plays an important role in statistical inference, which consider the properties of statistical procedures as the sample size increases. Geometrically, an approximation to a manifold is a local linearization by the tangent space. Thus, the tangent space of the manifold of the exponential family of distributions with progressively Type-II censored data is discussed in Section 2.
- The local linearization accounts only for local properties of a statistical model. It is necessary to investigate the Fisher metric tensors, affine connection, and α-connection of the manifold in order to study the global or large-scale properties of the model. Therefore, these important geometric quantities are studied in Section 3.
- As an application of the geometric quantities, the asymptotic expansions of the posterior density and the posterior Bayesian predictive density of the model are provided in Section 4.
- To illustrate the results presented in this paper, the Rayleigh distribution is considered as an example in Section 5. Moreover, Monte Carlo simulation results and a real data analysis are presented in Section 6 to illustrate the main results.

## 2. The Statistical Model and Tangent Space

In this paper, we adopt the Einstein summation convention, that is, if an index occurs both as a superscript and as a subscript in a single expression, then the summation over that index is implied. For a density function f(x;θ)∈F, let l(x;θ)=logf(x;θ), we introduce the following definitions (see [1,2] for more details):

- $g_{ij}\overset{\mathrm{def}}{=}E\left[\partial_{i}l\left(x;\theta \right)\partial_{j}l\left(x;\theta \right)\right]$: the Fisher metric tensors, the inverse of $g_{ij}$ is denoted by $g^{ij}$, where $\partial_{i}=\partial /\partial \theta^{i}$;
- $T_{ijk}\overset{\mathrm{def}}{=}E\left[\partial_{i}l\left(x;\theta \right)\partial_{j}l\left(x;\theta \right)\partial_{k}l\left(x;\theta \right)\right]$: the skewness tensor;
- $\Gamma_{ijk}\overset{\mathrm{def}}{=}E\left[\partial_{i}\partial_{j}l\left(x;\theta \right)\partial_{k}l\left(x;\theta \right)\right]$: the affine connection;
- $\Gamma_{ijk}^{\alpha }\overset{\mathrm{def}}{=}\Gamma_{ijk}+\frac{1-\alpha }{2}T_{ijk}$: the α-connection.

The −1-connection and 1-connection are said to be the *m*-connection and *e*-connection, denoted by $\Gamma_{ijk}^{m}$ and $\Gamma_{ijk}^{e}$, respectively. We also abbreviate some geometric terms by multiplication the metric tensors, i.e., $T_{i}=T_{ijk}g^{jk}$, $\Gamma_{ij}^{l}=\Gamma_{ijk}g^{kl}$, $\Gamma_{ij}^{\alpha ,l}=\Gamma_{ijk}^{\alpha }g^{kl}$.

Suppose that F={f(x,θ),θ∈Θ} is an exponential family of distributions (see, for example, Barndorff-Nielsen [16]) with density function

(1) $$\begin{array}{c} f\left(x,\theta \right)=\exp\left\{\sum_{i=1}^{\tau }\alpha^{i}\left(\theta \right)c_{i}\left(x\right)-\psi \left(\theta \right)\right\} \end{array}$$

and reliability function

(2) $$\begin{array}{c} R\left(x,\theta \right)=1-F\left(x,\theta \right)=\exp\left\{\sum_{i=1}^{\tau }\beta^{i}\left(\theta \right)d_{i}\left(x\right)-\phi \left(\theta \right)\right\}, \end{array}$$

where τ is the number of functions of the parameter vector θ, F(x,θ) is the cumulative distribution function, and ψ(θ) is the cumulant generating function defined as

$$\exp\left\{\psi \left(\theta \right)\right\}=\int \exp\left\{\sum_{i=1}^{\tau }\alpha^{i}\left(\theta \right)c_{i}\left(x\right)\right\}\mu \left(dx\right),$$

with $\alpha^{i}\left(\theta \right)$ and $\beta^{i}\left(\theta \right)$ are smooth functions of the parameter vector θ, and $c_{i}$ and $d_{i}$ are smooth functions of the random variable *x*. Here are two examples, the exponential and the Rayleigh distributions, of the members in the exponential family of distributions:

- Exponential distribution with density function
  f(x;λ)=exp{−λx+lnλ},x>0,λ>0,
  and reliability function
  R(x;λ)=exp{−λx},x>0,λ>0,
  we have τ=1 and the functions $\alpha^{1}\left(\lambda \right)=\beta^{1}\left(\lambda \right)=-\lambda$ and $c_{1}\left(x\right)=d_{1}\left(x\right)=x$, ψ(θ)=−lnλ and ϕ(θ)=0. The dimension of the parameter vector θ is k=1.
- Rayleigh distribution with density function
  (3) $$\begin{array}{c} f\left(x;\lambda \right)=\exp\left\{-\lambda x^{2}+\ln x+\ln2\lambda \right\},x>0,\lambda >0, \end{array}$$
  and reliability function
  $$R\left(x;\lambda \right)=\exp\left\{-\lambda x^{2}\right\},x>0,\lambda >0,$$
  We have τ=2 and the functions $\alpha^{1}\left(\lambda \right)=\beta^{1}\left(\lambda \right)=-\lambda$, $\alpha^{2}\left(\lambda \right)=1$, $\beta^{2}\left(\lambda \right)=0$, $c_{1}\left(x\right)=d_{1}\left(x\right)=x^{2}$, $c_{2}\left(x\right)=\ln x$, $d_{2}\left(x\right)=0$, ψ(θ)=−ln2λ and ϕ(θ)=0. The dimension of the parameter vector θ is k=1.

Consider the life-testing experiment with progressive Type-II censoring described in Section 1 with *n* items placed on the life testing experiment and *m* failures are planned to be observed, let the set of all admissible Type-II PCSs as

$$PC\left(m,n\right)=\left\{R=\left(R_{1},\cdots ,R_{m}\right)\in N_{0}^{m}|\sum_{i=1}^{m}R_{i}=n-m\right\},$$

where $N_{0}$ is the set of the non-negative integers. Under a given censoring scheme $R=\left(R_{1},\cdots ,R_{m}\right)\in PC\left(m,n\right)$, the set of progressively Type-II censored order statistics is denoted as $x_{m:n}^{R}=\left\{x_{1:m:n}^{R},\cdots ,x_{m:m:n}^{R}\right\}$. The PCS $R=(R_{1},\cdots ,R_{m})$ is prefixed prior to starting the life testing experiment.

Suppose the lifetime distribution of the items in the life testing experiment follows a distribution in the exponential family of distributions with density function in Equation (1), the joint density function of the observed data, $x_{m:n}^{R}$, can be expressed as [8,10]

(4) $$\begin{array}{ccc} L(x_{m:n}^{R};\theta ) & = & c\left(R\right)\prod_{r=1}^{m}f\left(x_{r:m:n}^{R},\theta \right){\left(1-F(x_{r:m:n}^{R},\theta )\right)}^{R_{r}}\\ & = & c\left(R\right)\prod_{r=1}^{m}\exp\left\{\sum_{i=1}^{\tau }\alpha^{i}\left(\theta \right)c_{i}\left(x_{r:m:n}^{R}\right)+\sum_{i=1}^{\tau }R_{r}\beta^{i}\left(\theta \right)d_{i}\left(x_{r:m:n}^{R}\right)-\psi \left(\theta \right)-R_{r}\phi \left(\theta \right)\right\}\\ & \overset{\mathrm{def}}{=} & c\left(R\right)\prod_{r=1}^{m}\exp\left\{\sum_{i=1}^{\tau }\theta^{i}e_{i}\left(x_{r:m:n}^{R}\right)-\varphi \left(\theta \right)\right\}\\ & = & c\left(R\right)\exp\left\{\sum_{r=1}^{m}\sum_{i=1}^{\tau }\theta^{i}e_{i}\left(x_{r:m:n}^{R}\right)-m\varphi \left(\theta \right)\right\}, \end{array}$$

where $\theta^{i}e_{i}\left(x_{r:m:n}^{R}\right)\overset{\mathrm{def}}{=}\alpha^{i}\left(\theta \right)c_{i}\left(x_{r:m:n}^{R}\right)+R_{r}\beta^{i}\left(\theta \right)d_{i}\left(x_{r:m:n}^{R}\right)$, $\varphi \left(\theta \right)\overset{\mathrm{def}}{=}\psi \left(\theta \right)+R_{r}\phi \left(\theta \right),$ and

$$c\left(R\right)=n\left(n-R_{1}-1\right)\cdots \left(n-R_{1}-R_{2}-\cdots -R_{m-1}-m+1\right)$$

is the normalizing constant. By defining a new random variables

$$x_{i;r:m:n}^{R}=e_{i}\left(x_{r:m:n}^{R}\right),$$

the joint density function in Equation (4) can be expressed as

$$\begin{array}{c} L\left(x_{m:n}^{R};\theta \right)=c\left(R\right)\exp\left\{\sum_{r=1}^{m}\sum_{i=1}^{\tau }\theta^{i}x_{i;r:m:n}^{R}-m\varphi \left(\theta \right)\right\}. \end{array}$$

The parameter θ of this form is called the natural parameter of the joint density function of the exponential family of distributions with progressive Type-II censoring.

The tangent vector $T_{\theta }$ of the manifold of the function $L(x_{m:n}^{R};\theta )$ is spanned by the vectors $\partial_{i}=\partial /\partial \theta^{i}$, and the set $\left\{\partial_{i}\right\}$ is called the natural basis associated with the coordinate system θ. Let

$$l\left(x_{m:n}^{R};\theta \right)=\log\left[L(x_{m:n}^{R};\theta )\right]=\log c\left(R\right)+\sum_{r=1}^{m}\sum_{i=1}^{\tau }\theta^{i}e_{i}\left(x_{r:m:n}^{R}\right)-m\varphi \left(\theta \right),$$

and the set

$$T_{\theta }^{\left(1\right)}=\left\{A\left(x_{m:n}^{R}\right)|A\left(x_{m:n}^{R}\right)=span\left\{\partial_{i}l\left(x_{m:n}^{R};\theta \right)\right\}\right\}$$

be the linear space of random variables spanned by $\partial_{i}l\left(x_{m:n}^{R};\theta \right)$. The space $T_{\theta }^{\left(1\right)}$ is called the 1-representation of the tangent space with progressively Type-II censored data. Here, the basis $\partial_{i}l\left(x_{m:n}^{R};\theta \right)$ of the 1-representation is given by

(5) $$\partial_{i}l\left(x_{m:n}^{R};\theta \right)=\sum_{r=1}^{m}e_{i}\left(x_{r:m:n}^{R}\right)-m\partial_{i}\varphi \left(\theta \right),$$

and the second and the third order derivatives of $l(x_{m:n}^{R};\theta )$ are given by

(6) $$\partial_{i}\partial_{j}l\left(x_{m:n}^{R};\theta \right)=-m\partial_{i}\partial_{j}\varphi \left(\theta \right),\;\;\;\partial_{i}\partial_{j}\partial_{k}l\left(x_{m:n}^{R};\theta \right)=-m\partial_{i}\partial_{j}\partial_{k}\varphi \left(\theta \right).$$

## 3. The α-Connections of Manifold Model

In this section, we investigate the α-connection of the manifold of the statistical model for the exponential family of distributions with progressively Type-II censored data. From Equation (4), the normalization factor φ(θ) can be defined as

$$\varphi \left(\theta \right)=\frac{1}{m}\log\int c\left(R\right)\exp\left\{\sum_{r=1}^{m}\sum_{i=1}^{\tau }\theta^{i}e_{i}\left(x_{r:m:n}^{R}\right)\right\}\mu \left(dx_{r:m:n}^{R}\right).$$

Since the function under the integral is assumed to be continuously differentiable, the order of integration and differentiation can be switched, and hence, the first three derivatives of the function φ(θ) with respect to the natural parameter $\theta^{i}$ are given by

(7) $$\begin{array}{ccc} \partial_{i}\varphi \left(\theta \right) & = & \frac{1}{m}\sum_{r=1}^{m}E_{L}\left[e_{i}\left(x_{r:m:n}^{R}\right)\right], \end{array}$$

(8) $$\begin{array}{ccc} \partial_{i}\partial_{j}\varphi \left(\theta \right) & = & \frac{1}{m}E_{L}\left[\left(\sum_{r=1}^{m}e_{i}\left(x_{r:m:n}^{R}\right)-m\partial_{i}\varphi \left(\theta \right)\right)\left(\sum_{r=1}^{m}e_{j}\left(x_{r:m:n}^{R}\right)-m\partial_{j}\varphi \left(\theta \right)\right)\right]\\ & = & \frac{1}{m}E_{L}\left[\partial_{i}l\left(x_{m:n}^{R};\theta \right)\partial_{j}l\left(x_{m:n}^{R};\theta \right)\right], \end{array}$$

(9) $$\begin{array}{ccc} \partial_{i}\partial_{j}\partial_{k}\varphi \left(\theta \right) & = & \frac{1}{m}E_{L}[\left(\sum_{r=1}^{m}e_{i}\left(x_{r:m:n}^{R}\right)-m\partial_{i}\varphi \left(\theta \right)\right)\left(\sum_{r=1}^{m}e_{j}\left(x_{r:m:n}^{R}\right)-m\partial_{j}\varphi \left(\theta \right)\right)\\ & & \times \left(\sum_{r=1}^{m}e_{k}\left(x_{r:m:n}^{R}\right)-m\partial_{k}\varphi \left(\theta \right)\right)]\\ & = & \frac{1}{m}E_{L}\left[\partial_{i}l\left(x_{m:n}^{R};\theta \right)\partial_{j}l\left(x_{m:n}^{R};\theta \right)\partial_{k}l\left(x_{m:n}^{R};\theta \right)\right], \end{array}$$

where the expectations $E_{L}\left[\cdot \right]$ are taken with respect to the joint density function in Equation (4). The derivatives in Equations (7)–(9) can be considered as the expected value, the covariance and the third-order central moments of $\sum_{r=1}^{m}e_{i}\left(x_{r:m:n}^{R}\right)$, respectively. The derivative in Equation (7) can also be obtained from the condition

$$E_{L}\left[\partial_{i}l\left(x_{m:n}^{R};\theta \right)\right]=0,$$

that is

$$0=\partial_{i}\int L\left(x_{m:n}^{R};\theta \right)\mu \left(dx_{m:n}^{R}\right)=\int \partial_{i}L\left(x_{m:n}^{R};\theta \right)\mu \left(dx_{m:n}^{R}\right)=E_{L}\left[\partial_{i}l\left(x_{m:n}^{R};\theta \right)\right].$$

The derivatives in Equations (8) and (9) can be obtained by calculating, respectively,

$$E_{L}\left[\partial_{i}\partial_{j}l\left(x_{m:n}^{R};\theta \right)\right]\;\;\;\;\mathrm{and}\;\;\;\;E_{L}\left[\partial_{i}\partial_{j}\partial_{k}l\left(x_{m:n}^{R};\theta \right)\right].$$

Equations (8) and (9) show that the (i,j) element of the metric tensors is given by

$$g_{ij}\left(\theta \right)=E_{L}\left[\partial_{i}l\left(x_{m:n}^{R};\theta \right)\partial_{j}l\left(x_{m:n}^{R};\theta \right)\right]=m\partial_{i}\partial_{j}\varphi \left(\theta \right),$$

the (i,j,k) element of the skewness tensor is given by

$$T_{ijk}\left(\theta \right)=E_{L}\left[\partial_{i}l\left(x_{m:n}^{R};\theta \right)\partial_{j}l\left(x_{m:n}^{R};\theta \right)\partial_{k}l\left(x_{m:n}^{R};\theta \right)\right]=m\partial_{i}\partial_{j}\partial_{k}\varphi \left(\theta \right),$$

and the (i,j,k) element of the affine connection is given by

$$\Gamma_{ijk}\left(\theta \right)=E_{L}\left[\partial_{i}\partial_{j}l\left(x_{m:n}^{R};\theta \right)\partial_{k}l\left(x_{m:n}^{R};\theta \right)\right]=-m\partial_{i}\partial_{j}\varphi \left(\theta \right)E_{L}\left[\partial_{k}l\left(x_{m:n}^{R};\theta \right)\right]=0.$$

Therefore, based on the joint density function $L(x_{m:n}^{R};\theta )$, the α-connection of the manifold of an exponential family of distributions is given by

$$\Gamma_{ijk}^{\alpha }\left(\theta \right)=\frac{(1-\alpha )m}{2}\partial_{i}\partial_{j}\partial_{k}\varphi \left(\theta \right),$$

which means that the natural parameter θ is 1-affine, i.e., $\Gamma_{ijk}=0$. Based on the information carried by the joint density function in Equation (4), we can obtain the following results.

**Theorem** **1.** *The metric tensors and the α-connection of the exponential family of distributions are given by*

$$\begin{array}{ccc} g_{ij}\left(\theta \right) & = & m\partial_{i}\partial_{j}\varphi \left(\theta \right)\\ and\Gamma_{ijk}^{\alpha }\left(\theta \right) & = & \frac{(1-\alpha )m}{2}\partial_{i}\partial_{j}\partial_{k}\varphi \left(\theta \right), \end{array}$$

*respectively.*

From the α-connection, we can obtain the torsion and the Riemann-Christoffel curvature of the manifold. The torsion is represented by the torsion tensor whose components are given by [1,2]

$$S_{ijk}\left(\theta \right)=\Gamma_{ijk}\left(\theta \right)-\Gamma_{jik}\left(\theta \right),$$

which is a tensor anti-symmetric with respect to indices i,j. Note that the coefficients $\Gamma_{ijk}^{\alpha }\left(\theta \right)$ of the α-connections are symmetric with respect to the first two indices *i* and *j*, then the tensor $S_{ijk}\left(\theta \right)$ vanishes for any α-connection. This shows that the manifold of the statistical model of the exponential family of distributions with progressively Type-II censored data is torsion-free.

The Riemann-Christoffel curvature of the manifold can be obtained as [1,2]

$$R_{ijkm}=\left(\partial_{i}\Gamma_{jk}^{s}-\partial_{j}\Gamma_{ik}^{s}\right)g_{sm}+\left(\Gamma_{irm}\Gamma_{jk}^{r}-\Gamma_{jrm}\Gamma_{ik}^{r}\right),$$

where $\Gamma_{ij}^{k}=g^{km}\Gamma_{ijm}$. The Riemann-Christoffel curvature based on the α-connection is called the α-Riemann-Christoffel curvature and its tensor is given by

$$R_{ijkm}^{\alpha }=\left(\partial_{i}\Gamma_{jk}^{\alpha ,s}-\partial_{j}\Gamma_{ik}^{\alpha ,s}\right)g_{sm}+\left(\Gamma_{irm}^{\alpha }\Gamma_{jk}^{\alpha ,r}-\Gamma_{jrm}^{\alpha }\Gamma_{ik}^{\alpha ,r}\right),$$

where $\Gamma_{ij}^{\alpha ,k}=g^{km}\Gamma_{ijm}^{\alpha }$. The tangent space of the manifold is said to be α-flat if the α-Riemann-Christoffel curvature $R_{ijkm}^{\alpha }=0$. We can also obtain the α-covariant derivative and the Laplace operator based on the α-connection and the metric tensors.

In the above process for obtaining those geometric quantities, we only use the information from the joint density function $L(x_{m:n}^{R};\theta )$. There is, in fact, another kind of information in the progressively Type-II censored order statistics $x_{r:m:n}^{R}$(r=1,⋯,m). We can consider the marginal density function of the *r*-th progressively Type-II censored order statistic, $x_{r:m:n}^{R}$ (see, for example, Kamps and Cramer [17], Balakrishnan [18], Balakrishnan and Aggarwala [8], and Balakrishnan and Cramer [10])

(10) $$\begin{array}{c} f_{x_{r:m:n}^{R}}\left(x\right)=c_{r-1}\sum_{s=1}^{r}a_{s,r}f\left(x\right){\left(1-F\left(x\right)\right)}^{\gamma_{s}-1},x>0,r=1,\cdots ,m, \end{array}$$

where

$$\begin{array}{ccc} \gamma_{s} & = & n-s+1+\sum_{r=1}^{s-1}R_{r}\;\mathrm{for}\;s=1,\cdots ,m,\\ c_{r-1} & = & \prod_{s=1}^{r}\gamma_{s}\;\mathrm{for}\;r=1,\cdots ,m,\\ a_{s,r} & = & \prod_{k=1,k\neq s}^{r}\frac{1}{\gamma_{k}-\gamma_{s}}\;\mathrm{for}\;1\leq s\leq r\leq m\;\mathrm{with}\;a_{1,1}=1. \end{array}$$

Based on the marginal density in Equation (10), the expectations of the random variables $e_{i}\left(x_{r:m:n}^{R}\right)$(r=1,⋯,m) in Equation (5) can be obtained as

$$\begin{array}{ccc} h_{i,r}\left(\theta \right) & \overset{\mathrm{def}}{=} & E_{f}\left[e_{i}\left(x_{r:m:n}^{R}\right)\right]=\int e_{i}\left(x\right)f_{x_{r:m:n}^{R}}\left(x\right)\mu \left(dx\right)\\ & = & c_{r-1}\sum_{s=1}^{r}a_{s,r}\int e_{i}\left(x\right)\exp\left\{\sum_{i=1}^{\tau }\theta^{i}e_{i,\gamma_{s}}\left(x\right)-\varphi_{\gamma_{s}}\left(\theta \right)\right\}\mu \left(dx\right), \end{array}$$

where $\theta^{i}e_{i,\gamma_{s}}\left(x\right)-\varphi_{\gamma_{s}}\left(\theta \right)=\alpha^{i}\left(\theta \right)c_{i}\left(x\right)+\left(\gamma_{s}-1\right)\beta^{i}\left(\theta \right)d_{i}\left(x\right)-\psi \left(\theta \right)-\left(\gamma_{s}-1\right)\phi \left(\theta \right),$ the expectation $E_{f}\left[\cdot \right]$ is taken with respect to the density function in Equation (10). Suppose that the random variables $e_{i}\left(x\right)$(r=1,⋯,m) are independent, and let

$$h_{i}\left(\theta \right)=\sum_{r=1}^{m}h_{i,r}\left(\theta \right)=\sum_{r=1}^{m}\sum_{s=1}^{r}c_{r-1}a_{s,r}\int e_{i}\left(x\right)\exp\left\{\sum_{i=1}^{\tau }\theta^{i}e_{i,\gamma_{s}}\left(x\right)-\varphi_{\gamma_{s}}\left(\theta \right)\right\}\mu \left(dx\right),$$

we can obtain the following results.

**Theorem** **2.** *The Fisher metric tensors and the α-connection of the exponential family of distribution with progressively Type-II censored data are given by*

$$\begin{array}{ccc} {\tilde{g}}_{ij}\left(\theta \right) & = & g_{ij}\left(\theta \right)=m\partial_{i}\partial_{j}\varphi \left(\theta \right),\\ {\tilde{\Gamma }}_{ijk}^{\alpha }\left(\theta \right) & = & g_{ij}\left(\theta \right)\left(m\partial_{k}\varphi \left(\theta \right)-h_{k}\left(\theta \right)\right)+\frac{1-\alpha }{2}\prod_{l=i,j,k}^{}\left(h_{l}\left(\theta \right)-m\partial_{l}\varphi \left(\theta \right)\right), \end{array}$$

*respectively.*

**Proof.**  For the metric tensors, they can be obtained directly from the definition of $g_{ij}\left(\theta \right)$. For the affine connection, from $E\left[\partial_{i}l\left(x_{m:n}^{R};\theta \right)\right]=\sum_{r=1}^{m}E_{f}\left[e_{i}\left(x_{r:m:n}^{R}\right)\right]-m\partial_{i}\varphi \left(\theta \right)=h_{i}\left(\theta \right)-m\partial_{i}\varphi \left(\theta \right)$, Equations (7) and (8), we can obtain

$$\begin{array}{ccc} {\tilde{\Gamma }}_{ijk}\left(\theta \right) & = & E[\partial_{i}\partial_{j}l\left(x_{m:n}^{R};\theta \right)\partial_{k}l\left(x_{m:n}^{R};\theta \right)]\\ & = & -m\partial_{i}\partial_{j}\varphi \left(\theta \right)E\left[\partial_{k}l\left(x_{m:n}^{R};\theta \right)\right]\\ & = & g_{ij}\left(\theta \right)\left(m\partial_{k}\varphi \left(\theta \right)-h_{k}\left(\theta \right)\right). \end{array}$$

Then, the third-order tensor $T_{ijk}\left(\theta \right)$ can be specified as

$$\begin{array}{ccc} {\tilde{T}}_{ijk}\left(\theta \right) & = & E\left[\partial_{i}l\left(x_{m:n}^{R};\theta \right)\partial_{j}l\left(x_{m:n}^{R};\theta \right)\partial_{k}l\left(x_{m:n}^{R};\theta \right)\right]=\prod_{l=i,j,k}^{}\left(h_{l}\left(\theta \right)-m\partial_{l}\varphi \left(\theta \right)\right). \end{array}$$

□

## 4. Applications in Bayesian Predictive Inference and Asymptotic Expansions

In Bayesian inference for the exponential family of distributions, the parameter vector θ is considered as a random variable. Given a prior density function for θ, π(θ), the joint posterior density function of the exponential family of distributions with progressively Type-II censored data can be expressed as

(11) $$\begin{array}{c} f_{\pi }\left(\theta |x_{m:n}^{R}\right)=\frac{L\left(x_{m:n}^{R};\theta \right)\pi \left(\theta \right)}{\int L\left(x_{m:n}^{R};\theta \right)\pi \left(\theta \right)d\theta }, \end{array}$$

and the posterior Bayesian predictive distribution is given by

(12) $$\begin{array}{c} {\hat{f}}_{\pi }\left(x|x_{m:n}^{R}\right)=\int f\left(x;\theta \right)f_{\pi }\left(\theta |x_{m:n}^{R}\right)d\theta , \end{array}$$

where *x* is an unobserved set of observations to be predicted and it is independently distributed according to the same density f(x;θ)∈F. The predictive density $\hat{f}\left(x|\hat{\theta }\right)$ is called the plug-in density function or the estimative density function, where $\hat{\theta }=\hat{\theta }\left(x_{m:n}^{R}\right)$ is an estimate of θ based on the observed progressively Type-II censored sample $x_{m:n}^{R}$ (see, for example, Geisser [19]). Consider the Kullback-Leibler divergence as the loss function, the predictive distribution in Equation (12) is the best predictive distribution in the sense that it minimizes the Bayes risk defined as [20]

$$\int \pi \left(\theta \right)\int f\left(x_{m:n}^{R};\theta \right)\int f\left(x;\theta \right)\log\left(\frac{f(x;\theta )}{\hat{f}\left(x|x_{m:n}^{R}\right)}\right)\;\mu \left(dx\right)\;\mu \left(dx_{m:n}^{R}\right)\;d\theta .$$

The integral defined in the predictive density in Equation (12) can be difficult to integrate or the form is too complicated to be used in practice. In these situations, asymptotic or large-sample theory (see, for example, Barndorff-Nielsen and Cox [21]) can be considered. In this section, we adopt the metric tensors and the α-connection introduced in Section 2 and Section 3 to study the asymptotic expansions of the posterior joint density and the Bayesian predictive density of the exponential family of distributions with progressively Type-II censored data. A similar asymptotic expansion of Bayesian prediction based on a full sample can be found in Zhang et al. [14]. For simplicity, we only consider the information carried by the joint density function in Equation (4), a similar process can be applied for the situation where the information obtained from the joint density function in Equation (4) and the marginal density function in Equation (10) together.

**Theorem** **3.** *Given a prior distribution π(θ) for θ, the posterior distribution in Equation (11) can be expressed asymptotically as*

$$\begin{array}{ccc} & & f_{\pi }\left(\theta |x_{m:n}^{R}\right)\\ & = & \frac{\det(g_{ij}\left(\theta \right))}{{\left(2\pi \right)}^{k/2}}\exp\left\{-\frac{1}{2}g_{ij}\left(\hat{\theta }\right){\tilde{\theta }}^{i}{\tilde{\theta }}^{j}\right\}\left(1+\frac{1}{6}T_{ijk}\left(\hat{\theta }\right){\tilde{\theta }}^{i}{\tilde{\theta }}^{j}{\tilde{\theta }}^{k}+\left(\partial_{i}\log\pi \left(\hat{\theta }\right)\right){\tilde{\theta }}^{i}+o^{-1}\left(n\right)\right), \end{array}$$

*where ${\tilde{\theta }}^{i}=\theta^{i}-{\hat{\theta }}^{i}$ and $\hat{\theta }$ is an estimator of parameter set θ.*

**Proof.**  Using the Laplace method suggested by Nielsen and Cox [21], the posterior distribution can be expressed asymptotically as

$$\begin{array}{ccc} f_{\pi }\left(\theta |x_{m:n}^{R}\right) & = & \frac{\det\left(-\partial_{i}\partial_{j}l\left(x_{m:n}^{R};\hat{\theta }\right)\right)}{{\left(2\pi \right)}^{k/2}}\exp\left\{-\frac{1}{2}\left(-\partial_{i}\partial_{j}l\left(x_{m:n}^{R};\hat{\theta }\right)\right){\tilde{\theta }}^{i}{\tilde{\theta }}^{j}\right\}\\ & & \times \left(1+\frac{1}{6}\left(\partial_{i}\partial_{j}\partial_{k}l\left(x_{m:n}^{R};\hat{\theta }\right)\right){\tilde{\theta }}^{i}{\tilde{\theta }}^{j}{\tilde{\theta }}^{k}+\left(\partial_{i}\log\pi \left(\hat{\theta }\right)\right){\tilde{\theta }}^{i}+o^{-1}\left(n\right)\right). \end{array}$$

We have

$$-\partial_{i}\partial_{j}l\left(x_{m:n}^{R};\theta \right)=m\partial_{i}\partial_{j}\varphi \left(\theta \right)=g_{ij}\left(\theta \right),\;\;\;-\partial_{i}\partial_{j}\partial_{k}l\left(x_{m:n}^{R};\theta \right)=m\partial_{i}\partial_{j}\partial_{k}\varphi \left(\theta \right)=T_{ijk}\left(\theta \right),$$

which implies that

$$\begin{array}{ccc} & & f_{\pi }\left(\theta |x_{m:n}^{R}\right)\\ & = & \frac{\det(g_{ij}\left(\hat{\theta }\right))}{{\left(2\pi \right)}^{k/2}}\exp\left\{-\frac{1}{2}g_{ij}\left(\hat{\theta }\right){\tilde{\theta }}^{i}{\tilde{\theta }}^{j}\right\}\left(1-\frac{1}{6}T_{ijk}\left(\hat{\theta }\right){\tilde{\theta }}^{i}{\tilde{\theta }}^{j}{\tilde{\theta }}^{k}+\left(\partial_{i}\log\pi \left(\hat{\theta }\right)\right){\tilde{\theta }}^{i}+o^{-1}\left(n\right)\right). \end{array}$$

□

Based on the asymptotic expansion presented in Theorem 3, we can obtain the following result.

**Theorem** **4.** *Given a prior distribution π(θ) for θ, the predictive distribution in Equation (12) can be expressed asymptotically as*

$$\begin{array}{ccc} {\hat{f}}_{\pi }\left(x|x_{m:n}^{R}\right) & = & f\left(x;\hat{\theta }\right)+\frac{1}{2n}g^{ij}\left(\hat{\theta }\right)\left\{-\partial_{i}\partial_{j}\psi \left(\hat{\theta }\right)+\Gamma_{ij}^{m,l}\left(\hat{\theta }\right)\left(c_{l}\left(x\right)-\partial_{l}\psi \left(\hat{\theta }\right)\right)\right\}\\ & & +\frac{1}{n}\left(\partial_{i}\log\pi \left(\hat{\theta }\right)-\Gamma_{ij}^{e,j}\left(\hat{\theta }\right)\right)g^{il}\left(\hat{\theta }\right)\left(c_{l}\left(x\right)-\partial_{l}\psi \left(\hat{\theta }\right)\right)+o\left(n^{-1}\right). \end{array}$$

**Proof.**  The proof is similar to the proof of Theorem 2 in Komaki [3]. The proof can be completed by substituting $\partial_{i}\partial_{j}f\left(x;\hat{\theta }\right)$ and $\partial_{i}f\left(x;\hat{\theta }\right)$ with $-\partial_{i}\partial_{j}\psi \left(\hat{\theta }\right)$ and $c_{i}\left(x\right)-\partial_{i}\psi \left(\hat{\theta }\right)$, respectively. □

If the prior distribution π(θ) is the Jeffreys prior $\pi_{J}\left(\theta \right)\propto {\left|g_{ij}\right|}^{\frac{1}{2}}$, then from the relationship

$$\partial_{i}\log\pi \left(\theta \right)=\partial_{i}\log{\left|g_{ij}\left(\theta \right)\right|}^{\frac{1}{2}}=\frac{1}{2}\partial_{i}g_{ij}\left(\theta \right)g^{ij}\left(\theta \right)=\Gamma_{ij}^{j}\left(\theta \right)=\Gamma_{ij}^{e,j}\left(\theta \right)+\frac{1}{2}T_{i}\left(\theta \right),$$

we have

$$\partial_{i}\log\pi \left(\theta \right)-\Gamma_{ij}^{e,j}\left(\theta \right)=\frac{1}{2}T_{i}\left(\theta \right).$$

The following results can be immediately obtained.

**Corollary** **1.** *Given the Jeffreys prior $\pi_{J}\left(\theta \right)\propto {\left|g_{ij}\left(\theta \right)\right|}^{1/2}$, the posterior distribution in Equation (11) can be asymptotically expanded as*

$$\begin{array}{ccc} f_{\pi_{J}}\left(\theta |x_{m:n}^{R}\right) & = & \frac{\det(g_{ij}\left(\hat{\theta }\right))}{{\left(2\pi \right)}^{k/2}}\exp\left\{-\frac{1}{2}g_{ij}\left(\hat{\theta }\right){\tilde{\theta }}^{i}{\tilde{\theta }}^{j}\right\}(1+\frac{1}{6}T_{ijk}\left(\hat{\theta }\right){\tilde{\theta }}^{i}{\tilde{\theta }}^{j}{\tilde{\theta }}^{k}\\ & & +\left(\Gamma_{ij}^{e,j}\left(\hat{\theta }\right)+\frac{1}{2}T_{i}\left(\hat{\theta }\right)\right){\tilde{\theta }}^{i}+o^{-1}\left(n\right)), \end{array}$$

**Corollary** **2.** *Given the Jeffreys prior $\pi_{J}\left(\theta \right)\propto {\left|g_{ij}\left(\theta \right)\right|}^{1/2}$, the prediction (12) can be asymptotically expanded as*

$$\begin{array}{ccc} {\hat{f}}_{\pi_{J}}\left(x|x_{m:n}^{R}\right) & = & f\left(x;\hat{\theta }\right)-\frac{1}{2n}g^{ij}\left(\hat{\theta }\right)\left\{\partial_{i}\partial_{j}\psi \left(\hat{\theta }\right)+\Gamma_{ij}^{m,l}\left(\hat{\theta }\right)\left(c_{l}\left(x\right)-\partial_{l}\psi \left(\hat{\theta }\right)\right)\right\}\\ & & +\frac{1}{2n}T_{i}\left(\hat{\theta }\right)g^{il}\left(\hat{\theta }\right)\left(c_{l}\left(x\right)-\partial_{l}\psi \left(\hat{\theta }\right)\right)+o\left(n^{-1}\right). \end{array}$$

These results show that the predictive density function, when the sample size *n* approaches infinity, is the estimative density function in the asymptotic sense.

## 5. Illustration Example

The illustration of the geometric quantities for exponential distribution has been provided in the literature (see, for example, [12]). In this section, we use the Rayleigh distribution, a member of the exponential family of distributions, presented in Section 2 as an example to illustrate our results. Suppose that $x_{m:n}^{R}$ is the progressively Type-II censored order statistics form items with lifetimes follow the Rayleigh distribution with density function in Equation (3), then the joint density function of $x_{m:n}^{R}$ can be expressed as

$$\begin{array}{ccc} L(x_{m:n}^{R};\theta ) & = & c\left(R\right)\prod_{r=1}^{m}f\left(x_{r:m:n}^{R},\theta \right){\left[1-F(x_{r:m:n}^{R},\theta )\right]}^{R_{r}}\\ & = & c\left(R\right)2^{m}\exp\left\{\sum_{r=1}^{m}\sum_{i=1}^{\tau }\theta^{i}e_{i}\left(x_{r:m:n}^{R}\right)-m\varphi \left(\theta \right)\right\}, \end{array}$$

where ℓ=2. $\theta^{1}=\lambda$, $\theta^{2}=1$, $e_{1}\left(x_{r:m:n}^{R}\right)=-\left(1+R_{r}\right){\left(x_{r:m:n}^{R}\right)}^{2}$, $e_{2}\left(x_{r:m:n}^{R}\right)=\ln\left(x_{r:m:n}^{R}\right)$ and φ(θ)=ψ(θ)=−ln(λ). Let

$$\begin{array}{ccc} l(x_{m:n}^{R};\theta ) & = & \log L(x_{m:n}^{R};\theta )\\ & = & \log\left(c\left(R\right)2^{m}\right)+\sum_{r=1}^{m}\sum_{i=1}^{\tau }\theta^{i}e_{i}\left(x_{r:m:n}^{R}\right)-m\varphi \left(\theta \right). \end{array}$$

Then, the first three derivatives of the function $l(x_{m:n}^{R};\theta )$ can be obtained as

$$\begin{array}{ccc} \partial_{1}l\left(x_{m:n}^{R};\theta \right) & = & -\sum_{r=1}^{m}\left(1+R_{r}\right){\left(x_{r:m:n}^{R}\right)}^{2}+\frac{m}{\lambda },\\ \partial_{1}\partial_{1}l\left(x_{m:n}^{R};\theta \right) & = & -\frac{m}{\lambda^{2}},\\ \partial_{1}\partial_{1}\partial_{1}l\left(x_{m:n}^{R};\theta \right) & = & \frac{2m}{\lambda^{3}}. \end{array}$$

The maximum likelihood estimator (MLE) of the parameter λ can be derived as

$$\hat{\lambda }=\frac{m}{\sum_{r=1}^{m}\left(1+R_{r}\right){\left(x_{r:m:n}^{R}\right)}^{2}}.$$

We first consider the information carried by the joint density in Equation (4). The metric tensors have one element, that is,

(13) $$\begin{array}{c} g_{11}\left(\theta \right)=m\partial_{1}\partial_{1}\varphi \left(\theta \right)=\frac{m}{\lambda^{2}}. \end{array}$$

The skewness tensor can be written as

(14) $$\begin{array}{c} T_{111}\left(\theta \right)=m\partial_{1}\partial_{1}\partial_{1}\varphi \left(\theta \right)=-\frac{2m}{\lambda^{3}}. \end{array}$$

The affine connection and the α-connection can be obtained as

$$\begin{array}{ccc} \Gamma_{111}\left(\theta \right) & = & -m\partial_{1}\partial_{1}\varphi \left(\theta \right)E_{L}\left[\partial_{1}l\left(x_{m:n}^{R};\theta \right)\right]=0,\\ \Gamma_{111}^{\alpha }\left(\theta \right) & = & \frac{(1-\alpha )m}{2}\partial_{1}\partial_{1}\partial_{1}\varphi \left(\theta \right)=\frac{(\alpha -1)m}{\lambda^{3}}, \end{array}$$

respectively. The coefficients of the *m*-connection and *e*-connection are

$$\begin{array}{ccc} G_{11}^{m,1}\left(\theta \right) & = & \Gamma_{111}^{m}\left(\theta \right)g^{11}\left(\theta \right)=-2/\lambda ,\\ G_{11}^{e,1}\left(\theta \right) & = & \Gamma_{111}^{e}\left(\theta \right)g^{11}\left(\theta \right)=0, \end{array}$$

respectively. For Bayesian inference, we consider the Jeffreys prior for the parameter λ, i.e.,

$$\pi_{J}\left(\theta \right)\propto \sqrt{m}/\lambda ,$$

then the posterior distribution of λ is

$$\begin{array}{c} f_{\pi_{J}}\left(\theta |x_{m:n}^{R}\right)=\frac{\exp\left\{\sum_{r=1}^{m}\sum_{i=1}^{\tau }\theta^{i}e_{i}\left(x_{r:m:n}^{R}\right)-\left(m-1\right)\varphi \left(\theta \right)\right\}}{\int \exp\left\{\sum_{r=1}^{m}\sum_{i=1}^{\tau }\theta^{i}e_{i}\left(x_{r:m:n}^{R}\right)-\left(m-1\right)\varphi \left(\theta \right)\right\}d\theta }, \end{array}$$

which can be written as

$$\begin{array}{ccc} & & f_{\pi_{J}}\left(\theta |x_{m:n}^{R}\right)\\ & & =\frac{\det(g_{ij}\left(\hat{\theta }\right))}{{\left(2\pi \right)}^{k/2}}\exp\left\{-\frac{1}{2}g_{ij}\left(\hat{\theta }\right){\tilde{\theta }}^{i}{\tilde{\theta }}^{j}\right\}(1-\frac{1}{6}T_{ijk}\left(\hat{\theta }\right){\tilde{\theta }}^{i}{\tilde{\theta }}^{j}{\tilde{\theta }}^{k}\\ & & \;+\left(\Gamma_{ij}^{e,j}\left(\hat{\theta }\right)+\frac{1}{2}T_{i}\left(\hat{\theta }\right)\right){\tilde{\theta }}^{i}+o^{-1}\left(n\right))\\ & & =\frac{m}{{\hat{\lambda }}^{2}{\left(2\pi \right)}^{1/2}}\exp\left\{-\frac{m}{2{\hat{\lambda }}^{2}}{\left(\lambda -\hat{\lambda }\right)}^{2}\right\}\left(1+\frac{m}{3{\hat{\lambda }}^{3}}{\left(\lambda -\hat{\lambda }\right)}^{3}-\frac{1}{\hat{\lambda }}\left(\lambda -\hat{\lambda }\right)+o\left(n^{-1}\right)\right). \end{array}$$

Here, the predictive distribution is

$$\begin{array}{c} {\hat{f}}_{\pi_{J}}\left(x|x_{m:n}^{R}\right)=c\left(R\right)2^{m}\int \exp\left\{\sum_{r=1}^{m}\sum_{i=1}^{\tau }\theta^{i}e_{i}\left(x_{r:m:n}^{R}\right)-\theta^{1}x^{2}+\ln\left(x\right)-\left(m+1\right)\varphi \left(\theta \right)\right\}d\theta , \end{array}$$

which can be expanded asymptotically as

$$\begin{array}{ccc} {\hat{f}}_{\pi_{J}}\left(x|x_{m:n}^{R}\right) & = & f\left(x;\hat{\theta }\right)-\frac{1}{2n}g^{ij}\left(\hat{\theta }\right)\left\{\partial_{i}\partial_{j}\psi \left(\hat{\theta }\right)+\Gamma_{ij}^{m,l}\left(\hat{\theta }\right)\left(c_{l}\left(x\right)-\partial_{l}\psi \left(\hat{\theta }\right)\right)\right\}\\ & & +\frac{1}{2n}T_{i}\left(\hat{\theta }\right)g^{il}\left(\hat{\theta }\right)\left(c_{l}\left(x\right)-\partial_{l}\psi \left(\hat{\theta }\right)\right)+o\left(n^{-1}\right)\\ & = & f\left(x,\hat{\lambda }\right)-\frac{{\hat{\lambda }}^{2}}{2nm}\left(-\frac{1}{2{\hat{\lambda }}^{2}}-\frac{2}{\hat{\lambda }}\left(\frac{1}{2\hat{\lambda }}-x^{2}\right)\right)\\ & & +\frac{1}{2n}\left(-\frac{2m}{{\hat{\lambda }}^{3}}\right)\frac{{\hat{\lambda }}^{2}}{m}\frac{{\hat{\lambda }}^{2}}{m}\left(\frac{1}{2\hat{\lambda }}-x^{2}\right)+o\left(n^{-1}\right)\\ & = & f\left(x,\hat{\lambda }\right)+\frac{1}{4mn}+o\left(n^{-1}\right). \end{array}$$

In the following, we consider the information obtained from the marginal density function in Equation (10) and the joint density function in Equation (4) together. Notice that

$$\begin{array}{ccc} f_{x_{r:m:n}^{R}}\left(x\right) & = & c_{r-1}\sum_{s=1}^{r}a_{s,r}f\left(x\right){\left(1-F\left(x\right)\right)}^{\gamma_{s}-1}\\ & = & c_{r-1}\sum_{s=1}^{r}a_{s,r}2\lambda x\exp\left\{-\lambda \gamma_{s}{\left(x_{r:m:n}^{R}\right)}^{2}\right\},r=1,\cdots ,m. \end{array}$$

The *n*-order moment about the origin of the *r*-th progressively Type-II censored order statistic is given by

$$\begin{array}{c} E_{f}\left[{\left(x_{r:m:n}^{R}\right)}^{n}\right]=\int x^{n}f_{x_{r:m:n}^{R}}\left(x\right)\mu \left(dx\right)=c_{r-1}\sum_{s=1}^{r}a_{s,r}\lambda \frac{\Gamma (\frac{n}{2}+1)}{{\left(\lambda \gamma_{s}\right)}^{\frac{n}{2}+1}}, \end{array}$$

which implies

$$\begin{array}{c} E_{f}\left[\partial_{1}l\left(x_{m:n}^{R};\theta \right)\right]=-\sum_{r=1}^{m}\sum_{s=1}^{r}\left(1+R_{r}\right)c_{r-1}\frac{a_{s,r}}{\lambda \gamma_{s}^{2}}+\frac{m}{\lambda }. \end{array}$$

Thus, the affine connection is specified as

$${\tilde{\Gamma }}_{111}\left(\theta \right)=E_{f}\left[\partial_{1}\partial_{1}l\left(x_{m:n}^{R};\theta \right)\partial_{1}l\left(x_{m:n}^{R};\theta \right)\right]=\frac{m}{\lambda^{3}}\sum_{r=1}^{m}\sum_{s=1}^{r}\left(1+R_{r}\right)c_{r-1}\frac{a_{s,r}}{\gamma_{s}^{2}}-\frac{m^{2}}{\lambda^{3}}.$$

The metric tensor ${\tilde{g}}_{11}\left(\theta \right)$ and the skewness tensor ${\tilde{T}}_{111}\left(\theta \right)$ are the same as the expressions in Equations (13) and (14). The α-connection is reduces to

$$\begin{array}{c} {\tilde{\Gamma }}_{111}^{\alpha }\left(\theta \right)=\frac{m}{\lambda^{3}}\sum_{r=1}^{m}\sum_{s=1}^{r}\left(1+R_{r}\right)c_{r-1}\frac{a_{s,r}}{\gamma_{s}^{2}}-\frac{m^{2}}{\lambda^{3}}-\frac{(1-\alpha )m}{\lambda^{3}}. \end{array}$$

The coefficients of the *m*-connection and the *e*-connection are

$$\begin{array}{ccc} {\tilde{\Gamma }}_{11}^{m,1}\left(\theta \right) & = & \frac{1}{\lambda }\sum_{r=1}^{m}\sum_{s=1}^{r}\left(1+R_{r}\right)c_{r-1}\frac{a_{s,r}}{\gamma_{s}^{2}}-\frac{2+m}{\lambda },\\ \mathrm{and}\;{\tilde{\Gamma }}_{11}^{e,1}\left(\theta \right) & = & \frac{1}{\lambda }\sum_{r=1}^{m}\sum_{s=1}^{r}\left(1+R_{r}\right)c_{r-1}\frac{a_{s,r}}{\gamma_{s}^{2}}-\frac{m}{\lambda }, \end{array}$$

respectively. Therefore, based on the Jeffreys prior $\pi_{J}\left(\theta \right)\propto \sqrt{m}/\lambda$, the Bayesian predictive density function of the Rayleigh distribution with progressively Type-II censored data can be asymptotically expanded as

$$\begin{array}{ccc} {\hat{f}}_{\pi_{J}}\left(x|x_{m:n}^{R}\right) & = & f\left(x;\hat{\theta }\right)-\frac{1}{2n}{\tilde{g}}^{ij}\left(\hat{\theta }\right)\left\{\partial_{i}\partial_{j}\psi \left(\hat{\theta }\right)+{\tilde{\Gamma }}_{ij}^{m,l}\left(\hat{\theta }\right)\left(c_{l}\left(x\right)-\partial_{l}\psi \left(\hat{\theta }\right)\right)\right\}\\ & & +\frac{1}{2n}{\tilde{T}}_{i}\left(\hat{\theta }\right)g^{il}\left(\hat{\theta }\right)\left(c_{l}\left(x\right)-\partial_{l}\psi \left(\hat{\theta }\right)\right)+o\left(n^{-1}\right)\\ & = & f\left(x,\hat{\lambda }\right)+\frac{1}{4mn}\left(1+\left(2\hat{\lambda }x^{2}-1\right)\left(\sum_{r=1}^{m}\sum_{s=1}^{r}\left(1+R_{r}\right)\frac{c_{r-1}a_{s,r}}{\gamma_{s}^{-2}}-m\right)\right)\\ & & +o(n^{-1}). \end{array}$$

This shows that the predictive density function, with the increase of the sample size *n* and the observed sample size *m*, is the estimative density function in the asymptotic sense. The term

$$\frac{2\hat{\lambda }x^{2}-1}{4mn}\left(\sum_{r=1}^{m}\sum_{s=1}^{r}\left(1+R_{r}\right)\frac{c_{r-1}a_{s,r}}{\gamma_{s}^{-2}}-m\right)$$

can be considered the correction term due to the information carried by the density function in Equation (10).

## 6. Monte Carlo Simulation Study and Real Data Analysis

In this section, we present a Monte Carlo simulation study of the Bayesian prediction based on progressively Type-II censored data described in Section 4. We also present a real data analysis based on the progressive Type-II censored data discussed in the literature. In the Monte Carlo simulation study, we consider different sample sizes (n,m)=(10,30), (10, 35), (15, 40) and (20, 40) and three different censoring schemes:

$R_{1}$: $R_{1}=R_{2}=\cdots =R_{m-1}=0,R_{m}=n-m$;
$R_{2}$: $R_{1}=n-m,R_{2}=R_{3}=\cdots =R_{m}=0$;
$R_{3}$: $R_{1}=\cdots =R_{m-1}=1,R_{m}=n-2m+1.$

The progressively Type-II censored data, $x_{m:n}^{R}$, are generated based on the Rayleigh distribution in Equation (3) with parameter λ=2 for different sample sizes and censoring schemes. For the proposed Bayesian prediction (BP), we consider two different priors: (i) the Jeffreys prior $\pi_{J}\left(\theta \right)\propto \sqrt{m}/\lambda$; and (ii) the uniform prior $\pi_{I}$ on interval (0,3). For comparative purposes, we also consider the plug-in prediction (PP) approach in which the estimative density function $\hat{f}\left(x,\hat{\lambda }\right)$ is also considered. For the plug-in approach, the parameter is estimated by using the maximum likelihood method based on the simulated progressive Type-II censored sample $x_{m:n}^{R}$. The estimated biases and mean square errors (MSEs) of different prediction approaches for predicting the probability density at x=2.5 based on 10,000 simulations are presented in Table 1.

From Table 1, we observe that the performances of all prediction methods improve in terms of MSEs as the sample sizes *m* and *n* increase. In other words, the number of items being removed during the progressively Type-II censored experiment affects the performance of prediction. Moreover, we observe that the Bayesian prediction method with the Jeffreys prior can provide smaller biases and smaller MSEs compared to the plug-in prediction method in some cases.

To illustrate the practical applications of the approximate methods based on geometric quantities proposed in this paper, we analyze a real data set which contains the times to breakdown of an insulating fluid at 34 kV originally presented in Nelson [22] (Table 6.1). A progressively Type-II censored sample of size m=8 generated from the n=19 observations by Balakrishnan et al. [9] is analyzed here. The progressively censored sample and the progressive censoring scheme are presented in Table 2.

**Figure 1:**
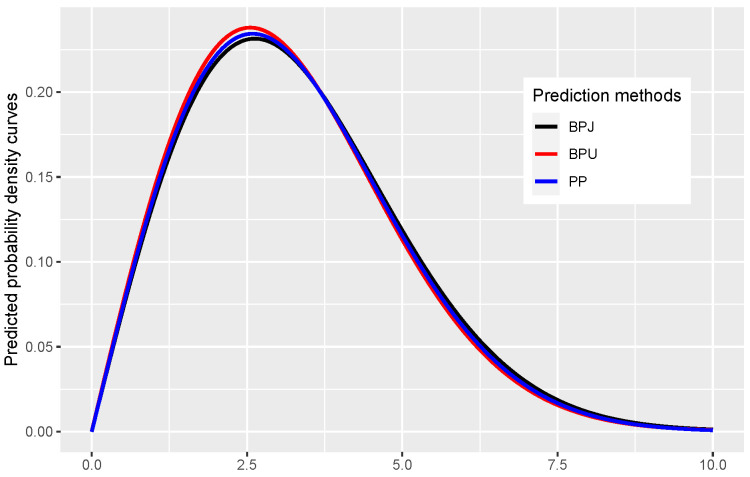
The predicted density curves of Rayleigh distribution obtained from the Bayesian prediction approach with Jeffreys prior (BPJ), with uniform prior to interval (0,3) (BPU), and the plug-in prediction (PP) approach based on the data presented in Table 2.

Suppose that we assume the lifetimes of the insulating fluid tested at 34 kV follow a Rayleigh distribution and we are interested in predicting the probability density, based on the progressive Type-II censored data presented in Table 2, the predicted density curves obtained from the plug-in prediction approach and the proposed Bayesian prediction approach with two different priors are presented in Figure 1. From Figure 1, we observe that the three prediction methods provide similar predicted density curves in this case. For instance, if we are interested in predicting density at x=2.8, based on the progressive Type-II censored data presented in Table 2, the predicted values of plug-in prediction density $\hat{f}\left(x,\hat{\lambda }\right)$ is 0.230, and the Bayesian prediction densities ${\hat{f}}_{\pi_{J}}\left(x|x_{m:n}^{R}\right)$ with Jeffreys prior $\pi_{J}$ and ${\hat{f}}_{\pi_{I}}\left(x|x_{m:n}^{R}\right)$ uniform prior $\pi_{I}$ are 0.229 and 0.232, respectively.

## 7. Conclusions

In this paper, we discussed the tangent space, affine connection, α-connection, torsion and Riemann-Christoffel curvature of statistical manifold induced by the exponential family of distributions. As applications of these geometric quantities, the asymptotic expansions of the Bayesian posterior distribution and prediction function with progressively Type-II censored data were discussed. The results showed that the asymptotic expansions are related to the geometric quantities. We also illustrated the main results by studying the Rayleigh distribution. Note that more theoretical results and applications of information geometry in reliability in addition to the main results of this paper can be found in the Ph.D. thesis [23].

## Figures and Tables

**Table 1:** Simulated biases and mean square errors (MSEs) of different prediction methods based on Rayleigh distribution with λ=2.

(m,n)	Schemes	PP $\hat{f}\left(x;\hat{\lambda }\right)$		BP ${\hat{f}}_{\pi_{J}}\left(x|x_{m:n}^{R}\right)$		BP ${\hat{f}}_{\pi_{I}}\left(x|x_{m:n}^{R}\right)$	
		Bias	MSE	Bias	MSE	Bias	MSE
	$R_{1}$	−0.045	0.032	−0.026	0.027	−0.038	0.035
(10,30)	$R_{2}$	0.035	0.031	0.024	0.023	−0.021	0.034
	$R_{3}$	−0.034	0.032	−0.025	0.026	0.013	0.037
	$R_{1}$	−0.030	0.029	−0.031	0.023	−0.016	0.033
(10,35)	$R_{2}$	0.025	0.027	−0.020	0.022	0.028	0.031
	$R_{3}$	−0.022	0.028	0.024	0.024	−0.027	0.032
	$R_{1}$	−0.023	0.019	0.021	0.022	0.022	0.025
(15,40)	$R_{2}$	−0.017	0.020	−0.013	0.018	−0.013	0.027
	$R_{3}$	0.024	0.021	0.021	0.019	−0.023	0.023
	$R_{1}$	−0.023	0.019	0.021	0.010	−0.026	0.014
(20,40)	$R_{2}$	0.018	0.014	0.015	0.008	0.024	0.015
	$R_{3}$	−0.013	0.017	−0.010	0.009	−0.017	0.012

**Table 2:** Progressively Type-II censored sample of the times to breakdown data on insulating fluid tested at 34 KV with n=19 and m=8 obtained from Balakrishnan et al. [9].

*r*	1	2	3	4	5	6	7	8
$x_{r:n}$	0.19	0.78	0.96	1.31	2.78	4.85	6.50	7.35
$R_{r}$	0	0	3	0	3	0	0	5
